# Attitudes Toward Gay Men and Lesbian Women Moderate Heterosexual Adults’ Subjective Stress Response to Witnessing Homonegativity

**DOI:** 10.3389/fpsyg.2019.02948

**Published:** 2020-01-10

**Authors:** Hunter Hahn, Ilana Seager van Dyk, Woo-Young Ahn

**Affiliations:** ^1^Department of Psychology, The Ohio State University, Columbus, OH, United States; ^2^Department of Psychology, Seoul National University, Seoul, South Korea

**Keywords:** minority stress, ally, homonegativity, attitudes toward sexual minorities, sexual minorities

## Abstract

Minority stress theory posits that members of a stigmatized group, such as sexual minorities (lesbian, gay, bisexual, and other non-heterosexual individuals), are particularly subject to ill effects of minority-specific events (stressors), including overt homonegativity. Although adverse effects of homonegativity are well documented for sexual minorities, little is known about effects of witnessing homonegativity on heterosexual individuals. As a growing number of heterosexual individuals hold accepting views of sexual minority individuals, some detrimental effects of homonegativity may extend to heterosexual individuals. For example, prior studies demonstrate that when racial majority-group members witness discrimination against minority-group members, they may experience stress response, particularly if they hold positive attitudes toward the minority-group. In this experimental study, 263 heterosexual adults (*M*age = 34.47 years, *SD* = 9.67, 51.7% female) were randomized to either witness homonegativity or to a control condition. Participants rated subjective stress on a 0–100 visual analogue scale both immediately before and after the film-based induction. Participants also completed a measure of their attitudes toward gay men and lesbian women. Moderation analyses indicated that participants who were more accepting of gay men and lesbian women experienced greater stress after the induction than those with less accepting views. Implications and limitations of these findings are discussed.

## Introduction

Effects of stigma based on sexual orientation are well documented among sexual minorities (lesbian, gay, bisexual, and other non-heterosexual individuals; Meyer, 2003; Hatzenbuehler, 2009). For example, sexual minorities experience higher rates of both internalizing (e.g., anxiety and depression) and externalizing (e.g., substance abuse) psychopathology/risk behavior compared to heterosexual individuals (Conron et al., 2010; Marshal et al., 2011, 2012; Mustanski et al., 2011). The minority stress model proposes that these disparities result, in part, from unique stressors (e.g., discrimination and stigma) experienced by sexual minorities, jointly termed minority stress (Meyer, 2003). Minority stress may result from pervasive homonegativity, which we use here to describe any prejudicial or stigmatizing behavior directed toward sexual minorities, similar to the more colloquially used term homophobia (see Lottes and Grollman, 2010; Herek and McLemore, 2013). Although its original and most extensive use is to explain health disparities among sexual minorities, the minority stress model has since been extended to other stigmatized groups (e.g., Hendricks and Testa, 2012; Sikorski et al., 2015).

Importantly, this model emphasizes that effects of minority stress exposure depend on identification as a member of a stigmatized group (Meyer, 2003). In this case, those who identify as sexual minorities are susceptible to long-term adverse outcomes as a result of sexual orientation-related discrimination, whereas heterosexual individuals are not exposed to such stressors and thus are less vulnerable to the adverse mental health outcomes associated with minority stress. Thus, both sexual minority and heterosexual individuals may experience the same objective event (e.g., witnessing homonegativity), yet only sexual minority individuals would be at risk for developing adverse outcomes as a result (Meyer, 2003).

Understandably then, work that examines effects of homonegativity appears to focus on sexual minorities, and less is known about effects of homonegativity on heterosexual individuals. While one study found that heterosexual women acted more inclusively after witnessing ostracization of a gay man (Salvati et al., 2019), little is known about behavioral or stress responses to homonegativity. However, it is possible that heterosexual individuals who have accepting views of sexual minorities may experience a stress response after witnessing homonegativity (though likely differently and to a lesser extent than for sexual minorities). The human stress response is expansive and includes physiological/immunological (Porges, 2001; Dickerson and Kemeny, 2004; Marsland et al., 2017), cognitive (Staal, 2004; Lupien et al., 2009), and emotional changes (Lazarus, 2006; Okon-Singer et al., 2015) that interact in a social context (e.g., Meyer, 2003; von Dawans et al., 2012). Although little is known about heterosexual individuals’ stress responses to witnessing homonegativity, some insight may be drawn from research examining White individuals’ responses to witnessing racism.

Lickel et al. (2011) describe group-based emotional responses to discrimination, which occur when individuals recognize present-day or historical misdoings of their social groups. For example, White individuals may feel shame when witnessing other White individuals acting in a racist manner (Lickel et al., 2011). Prior work has found that racism may create psychological burden among White individuals (Todd et al., 2011), such as negative affect (e.g., guilt and shame) or cognitions (Spanierman and Heppner, 2004; Spanierman et al., 2006; Todd et al., 2011). Importantly, majority-group members who show more empathy toward racial minority members may experience unique negative emotional reactions, including guilt and hopelessness in combating racism (Spanierman et al., 2006). Similarly, White individuals with more positive views about diversity and inclusion of Black individuals reported more subjective negative valance and a greater physiological stress response after viewing an anti-diversity discussion, compared to those with less positive views about diversity (Schmader et al., 2011).

While there remains variability in heterosexual individuals’ attitudes toward sexual minorities, the 21st century has seen major advances in legal rights and acceptance of sexual minority individuals (Smith et al., 2014; Mendos, 2019). With growing acceptance of sexual minorities, more heterosexual people may be affected by witnessing homonegativity. Some heterosexual individuals, such as allies, may feel pressure to confront homonegativity when they witness it (Lapointe, 2015), yet others may be unsure of how to respond, potentially producing psychological distress (Ryan and Wessel, 2012). Still others, such as those who hold heterosexist beliefs and values, may experience little response to witnessing homonegativity.

Thus, there is reason to believe that *some* majority-group members may experience adverse effects when encountering discrimination. Though prior studies suggest that racial majority-group members’ subjective responses to witnessing racism/discrimination differ by attitudes toward minority-group members, this has not been experimentally examined among heterosexual individuals exposed to homonegativity. Therefore, it is unknown if these findings generalize to heterosexual individuals’ experiences after witnessing homonegativity. With little experimental work examining exposure to homonegativity among either sexual minorities or heterosexual people, specific components of potential stress or behavioral responses to homonegativity are unknown (e.g., distinctions between subjective, physiological, and emotional/behavioral responses to stress). Thus, at this early stage, it may be useful to consider heterosexual individuals’ exposure to homonegativity more generally as a potential acute stressor.

The general stress literature demonstrates adverse effects of acute stress on behavioral processes such as increased risky decision-making (Porcelli and Delgado, 2009; Mather and Lighthall, 2012). Similarly, acute stress resulting from viewing emotionally upsetting stimuli (e.g., violence) impairs working memory (Qin et al., 2009), and perceived stress after acute stress induction is associated with reduced self-control in health-related decision-making (Maier et al., 2015). Therefore, even acute exposure to homonegativity, if it produces a stress response, may increase vulnerability to adverse outcomes for heterosexual individuals.

Taken together, heterosexual individuals’ responses to homonegativity may differ significantly based on their attitudes toward sexual minorities, and a growing number of heterosexual people who hold more positive attitudes may be particularly affected by witnessing homonegativity compared to those with neutral or negative views. In line with a minority stress model (Meyer, 2003) and work on majority-group response to discrimination (Lickel et al., 2011), we might expect that some heterosexual individuals – especially those who hold more positive views of sexual minorities – would experience an acute stress response upon witnessing homonegativity.

### Current Study and Hypotheses

To address gaps in current research, we experimentally examined effects of exposure to homonegativity on self-reported perceived stress, one component of the general stress response. We hypothesized that (1) individuals witnessing homonegativity would experience a stronger subjective stress response than those who saw a neutral film and (2) heterosexual participants with more accepting attitudes toward sexual minorities would experience greater subjective stress responses after exposure to homonegativity.

## Materials and Methods

### Participants

Any adults living in the United States and identifying as heterosexual were eligible for participation. Participants (*n* = 276) were recruited online through Amazon’s Mechanical Turk as part of a larger, unpublished study of minority stress effects on decision-making among sexual minorities. For more details on Mechanical Turk, see Buhrmester et al. (2011). Of those who completed the study, ten were removed from analyses due to failure on more than two of ten attention check questions (e.g., “Click strongly agree”), used to ensure data integrity. Three participants were removed for changing self-reported sexual orientation from pre-screening (from heterosexual to a non-heterosexual orientation), as the study’s focus was on individuals who identified as heterosexual on the day of the experiment. Thus, the final sample included 263 individuals (*M*age = 34.47 years, *SD* = 9.67, 51.7% female). Most participants identified as White (*n* = 178, 67.7%), followed by Asian (*n* = 33, 12.5%), Black (*n* = 25, 9.5%), multiracial (*n* = 12, 4.6%), Hispanic/Latino (*n* = 9, 3.4%), and another race (*n* = 6, 2.3%).

### Procedure

All procedures were approved by the local Institutional Review Board in accordance with standard ethical guidelines, and participants provided informed consent before beginning the study. Respondents received $10 for participating. Participants were randomized to either a neutral control condition (*n* = 128) or a homonegativity condition (*n* = 135). In addition to measures examined in the present study (described below), as part of the larger study participants also completed a measure of resistance to peer influence and past-month perceived stress before the experimental manipulation (Cohen et al., 1983; Steinberg and Monahan, 2007). Participants completed all questionnaires before the experimental manipulation, with the exception of the post-manipulation stress measure.

### Measures

#### Demographics

Demographic variables included age, biological sex (*male* or *female*), sexual orientation (*heterosexual*, *gay or lesbian*, *bisexual*, or *another sexual orientation*), and race/ethnicity (*Hispanic/Latino*, *African-American/Black*, *Asian/Pacific Islander*, *Caucasian/White*, or another race). Participants could pick multiple racial/ethnic identities if applicable.

#### Homonegativity Manipulation

A two-minute film was used to expose participants to witnessing homonegativity (Seager, 2016). This film was previously validated with sexual minority adults to induce a minority stress response (for more details on this stimulus, see Seager, 2016). The video comprises of several short clips taken from mass media sources such as local and cable news media, church sermons, and television shows. Each clip shows adults making homonegative or heterosexist comments (e.g., “Don’t employ gays in the military, education, health, or psychology”; “It’s Adam and Eve, not Adam and Steve”). A two-minute video depicting a walking tour of London, United Kingdom with ambient background noise was used in the neutral control condition.

#### Stress Response

Participants reported their current stress levels both immediately before and after watching the videos, using a 0–100 visual analogue scale (0 = “Not stressed at all,” 100 = “Extremely stressed;” Maier et al., 2015). This scale was previously used to examine perceived stress, one aspect of the psychophysiological stress response, with ratings on this scale showing association with stress-related brain activity and behavioral responses to stress (Maier et al., 2015).

#### Attitudes Toward Gay Men/Lesbian Women

Attitudes toward gay and lesbian individuals were assessed using the “Gay Male/Lesbian Social Norms/Morality” subscale of the Component Measure of Attitudes Toward Homosexuality (LaMar and Kite, 1998). This scale includes ten items assessing views of gay men and lesbian women in society (e.g., “Gay men and lesbians just can’t fit into our society” or “Gay men and lesbians endanger the institution of the family”). Participants rated items on a Likert-type scale from 1 (“Strongly Agree”) to 5 (“Strongly Disagree”). A mean score of the ten items was used in analysis, with mean scores ranging from 1 to 5. Higher scores indicate more accepting views. In the validation study, the minimum Cronbach’s alpha was 0.92.

#### Data Analysis

Data were analyzed using SPSS version 25 (IBM Corporation, Armonk, NY, United States) and the PROCESS SPSS macro version 3 (Hayes, 2018). Because this study is a secondary analysis of a larger study, an *a priori* power analysis was not conducted for the present analyses. Regression-based moderation with 5,000 bootstrap samples was used to assess the relationship between attitudes toward gay and lesbian individuals and stress among both the homonegativity group and neutral control group. Analyses used a change score (post-induction stress – pre-induction stress) as the outcome measure when indicated in the section “Results.” In the moderation model, condition served as the independent variable, stress change score served as the dependent variable, and attitudes toward gay men/lesbian women served as the moderator variable. Conditions were dummy coded as 0 (homonegativity condition) and 1 (neutral condition) and scores on the attitudes measure were mean centered before inclusion in the moderation model.

## Results

No data were missing, and no outliers were identified for post-induction stress or the attitudes toward gay men/lesbian women measure. Seven participants (2.7%) were identified as outliers on the pre-induction stress measure, with pre-induction stress ratings greater than 80. Because these scores represent realistically possible differences in perceived stress, and since stress measures were a within-subject factor, we chose to retain these individuals in analysis.

Groups did not differ on race, χ^2^(5) = 4.03, *p* = 0.54; sex, χ^2^(1) = 0.04, *p* = 0.84; age, *t*(261) = −0.22, *p* = 0.83, *d* = 0.03, or pre-induction stress *t*(261) = −0.82, *p* = 0.41, *d* = 0.1. Significant group differences in post-induction stress were observed. Change scores indicate that those in the homonegativity condition reported significant increases in stress (*M* = 11.51, *SD* = 19.90) compared to those in the neutral condition (*M* = −1.00, *SD* = 10.57), *t*(261) = 6.32, *p* < 0.001, *d* = 0.79. Overall, participants reported generally accepting views of gay men and lesbian women, *M* = 4.00, *SD* = 1.13, as indexed by average scores over the median possible value of the scale. There were no differences in attitudes toward gay men and lesbian women by group, *t*(261) = 0.53, *p* = 0.60, *d* = 0.07.

**FIGURE 1:**
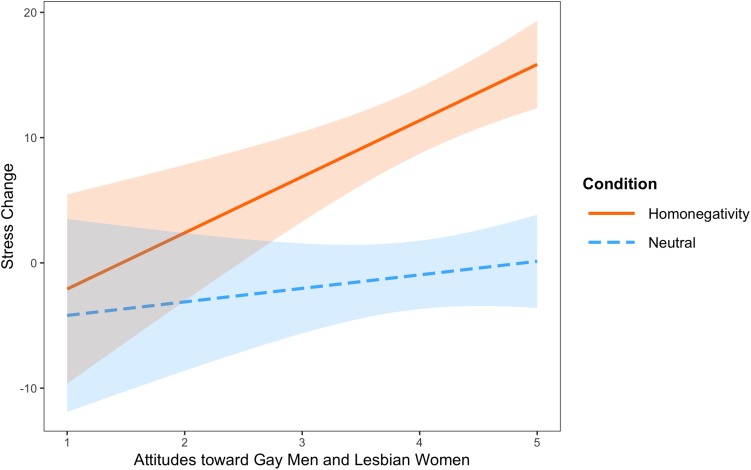
Interaction between stress condition and attitudes toward sexual minorities in predicting stress change. Stress was measured on a visual analogue scale from 0 to 100 (see text). Attitudes toward sexual minorities range from 1 to 5, with higher scores indicating more accepting attitudes. Shading represents 95% confidence intervals.

The overall model predicting change in stress was significant, *F*(3,259) = 18.99, *p* < 0.001, *R*^2^ = 0.42. The main effect of condition did not predict change in stress, *b* = 1.29, *t*(259) = 0.18, *p* = 0.86. Indeed, attitudes toward sexual minorities moderated the relationship between condition and change in stress, *b* = −3.40, *t*(259) = −1.99, *p* = 0.048. The moderation remained significant when controlling for age and sex, *p* = 0.05. As shown in Figure 1, exposure to videos of homonegative comments produced greater stress for those with positive attitudes toward gay men and lesbian women than for those with negative attitudes, *b* = 4.48, *t*(258) = 3.78, *p* = 0.0002. In contrast, exposure to neutral videos yielded no association between attitudes and stress, *b* = 1.08, *t*(258) = 0.87, *p* = 0.38.

## Discussion

We examined self-reported stress in heterosexual individuals after witnessing homonegativity. Those who were exposed to homonegative videos showed significant increases in self-reported perceived stress compared to those who viewed a neutral stimulus, even though the stimulus presented was not originally designed to produce a stress response among heterosexual individuals (Seager, 2016). Furthermore, those with more accepting views of gay and lesbian individuals showed an even stronger stress response compared to those with less accepting views, consistent with our hypothesis.

There may be several reasons why participants experienced a subjective stress response when witnessing homonegativity. From a minority stress perspective (Meyer, 2003), if a heterosexual individual holds positive views of sexual minorities and identifies as an ally, they may interpret homonegativity as an affront to their own identity and values, producing distress. More broadly, work on vicarious or collective guilt suggests that majority-group members may feel some personal responsibility for wrong-doing by others in their social group (Lickel et al., 2005; Wohl et al., 2010) – in this case, heterosexual individuals who did not themselves engage in homonegativity may still feel responsibility when witnessing other (presumed) heterosexual individuals acting homonegativity. As described previously, collective guilt of this nature can vary by individual characteristics, including attitudes toward the discriminated minority group (e.g., Lickel et al., 2011).

When considering implications of these findings, it is important to acknowledge the often cascading nature of homonegativity when experienced by sexual minorities. Minority stress theory distinguishes between two types of stressors: *distal* stressors, such as discrimination, which are external events that occur *to* a minority individual; and *proximal* stressors, such as internalized homonegativity, which occur *within* individuals (Meyer, 2003). Our experimental induction served as an objective, distal stressor. In contrast to sexual minorities, heterosexual individuals exposed to distal minority stress events are unlikely to experience more proximal stress reactions, which may drive health disparities among sexual minorities in the long-term (Meyer, 2003). Indeed, empirical findings suggest that proximal minority stress processes, such as internalized homonegativity, can mediate the relationship between distal minority stress exposure (e.g., discrimination) and mental health outcomes (Burks et al., 2015). Although heterosexual individuals likely experience only distal stress when witnessing homonegativity, and thus likely do not experience chronic stress related to homonegativity, an acute stress response may lead to cognitive, emotional, and behavioral changes relevant to heterosexual people.

These initial findings suggest that holding more positive attitudes toward gay and lesbian individuals opens heterosexual individuals up to additional psychological burdens when witnessing homonegativity. While we did not directly assess ally identity, heterosexual allies to the broader lesbian, gay, bisexual, transgender, and queer community may be particularly affected by witnessing homonegativity. Despite this finding, it is important to note that benefits of being an ally to sexual minority individuals are also well-documented (see Jones et al., 2014; Rostosky et al., 2015). For sexual minorities themselves, support from allies is consistently linked with improved outcomes (Goodenow et al., 2006; Walls et al., 2009). Thus, with a more detailed understanding of heterosexual individuals’ experience of witnessing homonegativity, trainings aimed at developing and engaging allies to sexual minorities may be refined and enhanced.

Importantly, stress of the nature shown here may potentially lead to both positive and negative outcomes. Past studies found that acute stress is associated with diminished self-control (Maier et al., 2015), reduced attention and working memory (Olver et al., 2015), and antisocial decision-making (Bendahan et al., 2017). However, other studies have shown that general acute stress may increase prosocial behavior by modulating empathy (Tomova et al., 2017). Similarly, individuals witnessing ostracization may be more likely to engage in prosocial behavior toward the ostracized individual, including ostracized gay men (Paolini et al., 2017; Salvati et al., 2019). Further, negative emotions such as guilt motivate some White individuals to engage in anti-racist activism (Case, 2012; Spanierman et al., 2012) or heterosexual individuals to become allies to sexual minorities (Brooks and Edwards, 2009; Asta and Vacha-Haase, 2012). However, others suggest that positive affect is a primary motivation for heterosexual ally engagement, contrasting with White anti-racist activities (Grzanka et al., 2015). In addition, specific emotional responses (e.g., anger, shame, and guilt) may differentially predict engagement vs. disengagement both in relation to discrimination and behavioral change more broadly (Iyer et al., 2007; Lickel et al., 2011, 2014), so it is unclear whether stress of the nature described here would lead to ally engagement or disengagement. These widely differing outcomes highlight the need to further examine the complex responses possible when heterosexual individuals witness homonegativity.

Our findings may have implications for future experimental research on heterosexual adults’ exposure to homonegativity. Researchers should consider differential effects of exposure to homonegativity among heterosexual individuals in domains beyond subjective stress, such as in decision-making, mental health outcomes, and health risk behaviors. In addition, minority stress researchers could further investigate how experiences of homonegativity differ between heterosexual individuals and sexual minorities. The present research found that heterosexual individuals experienced subjective stress responses to objective discrimination events; however, such responses may vary between sexual minorities and heterosexual individuals. For example, differences might emerge for specific emotional responses, cognitive appraisals of stressors, and/or psychophysiological stress responses. Identifying similarities and differences between the stress response of heterosexual individuals and sexual minorities is crucial for health disparity research (e.g., Hatzenbuehler, 2009).

### Limitations

Several limitations should be considered when interpreting our results. First, self-reports capture only one aspect of stress response. Other components of stress reactivity, including physiological markers and stress hormones, often yield complementary information (see e.g., Zisner and Beauchaine, 2016; Lovallo and Buchanan, 2017). In addition, we did not assess social desirability, which could have affected participants’ self-reported attitudes toward gay men and lesbian women. However, the online, anonymous nature of the study likely reduced such effects. Our dataset included only composite scores of the attitudes measure. Thus, without individual item scores, were unable to calculate Cronbach’s alpha of the attitudes toward gay men/lesbian women measure in the present sample. However, the original validation study showed strong reliability of this measure (LaMar and Kite, 1998). Next, the Attitudes toward Homosexuality measure included only a subset of sexual minorities, namely gay and lesbian individuals, rather than sexual minorities more broadly. Findings may differ based on a more inclusive measure (e.g., including attitudes toward bisexual individuals). In the present study, other variables may account for the findings, such as general reaction to emotional stimuli rather than a specific reaction to witnessing homonegativity. Indeed, there is a possibility that the response demonstrated here may not be specific to witnessing homonegativity, and could be common to other experiences producing negative valance. However, our findings show that those with more negative attitudes toward sexual minorities showed little response to witnessing homonegativity (see Figure 1). One would not expect these individuals to be less capable of experiencing any negative affect, but that they show less response to specifically to witnessing homonegativity, as shown here. Thus, although we would not hypothesize that attitudes toward sexual minorities moderate response to a general negative stimulus, future experiments could include additional comparison groups to empirically examine this possibility.

## Conclusion and Future Directions

Our findings suggest that some individuals, particularly those who hold positive views toward sexual minorities, report significant subjective stress upon witnessing homonegativity. There remains considerable room for expanding these findings to examine specific components of the stress response and its relation to behavioral change. Future work could examine additional stress responses to heterosexual individuals’ experience of homonegativity, such as psychophysiological responding, emotional changes, and behavioral effects. For example, psychophysiological response of White individuals after witnessing discrimination against Black individuals were moderated by their views of diversity (Schmader et al., 2011). Similarly, specific emotional responses (e.g., guilt, shame, and anger) to witnessing discrimination are distinguishable (Lickel et al., 2005) and may differentially motivate behavior either by engaging or disengaging individuals in combatting prejudice (Lickel et al., 2011); thus, future studies could examine specific behavioral responses to witnessing homonegativity. Ultimately, this work could inform programs developed to engage heterosexual allies in combatting homonegativity and build upon existing work on majority-group identity and response to discrimination of out-group members (e.g., Wohl et al., 2010).

Although this brief report only scratches the surface of heterosexual individuals’ response to witnessing homonegativity, our study adds to a growing literature highlighting detrimental effects of homonegativity and discrimination based on sexual orientation. As acceptance of sexual minorities continues to grow in the general population, detrimental effects of homonegativity may affect an increasing number of individuals, including heterosexual individuals. We hope that our findings inform both future experimental research on minority stress, as well as community-based intervention and prevention efforts that engage heterosexual individuals in reducing sexual orientation-related stigma.

## Data Availability Statement

The datasets generated for this study are available on request to the corresponding authors.

## Ethics Statement

The studies involving human participants were reviewed and approved by Institutional Review Board, The Ohio State University. The patients/participants provided their informed consent to participate in this study.

## Author Contributions

HH collected the data, conducted data analysis and literature reviews, and wrote the first draft of the manuscript. All authors conceptualized the study, assisted with data analysis, provided crucial feedback and edits on the final version of the manuscript, and approved the final version of the manuscript.

## Conflict of Interest

The authors declare that the research was conducted in the absence of any commercial or financial relationships that could be construed as a potential conflict of interest.
